# Taking responsibility

**DOI:** 10.3205/zma001548

**Published:** 2022-04-14

**Authors:** Sigrid Harendza

**Affiliations:** 1 Universitätsklinikum Hamburg-Eppendorf, III. Medizinische Klinik, Hamburg, Germany

## Editorial

You can take or have responsibility. But you can also carry it, which sounds more like a burden, or hand it over – when it becomes too heavy? You can also refuse responsibility if someone wants to give it to you, but you yourself think that you cannot carry it or do not want to take it. Sounds kind of tricky, and yet “responsibility” is the competence considered most important for beginning residents by physicians from different specialties and medical schools [1]. Many activities, which for the most part may only be performed by medical students under supervision according to the Medical Licensing Regulations, must be entrusted to physicians in training from the first day of work, which at the same time means they take the responsibility. In the case of nurses, for example, it has been shown that the responsibility they receive when they start work is greater than they can bear at that time, which leads to increased stress [2]. 

However, taking responsibility does not begin with the first day of medical work. It begins with the first day of undergraduate training. And actually even before, because taking responsibility means recognizing the goal and scope of a task and using one's own knowledge and skills and a self-reflective attitude to work for its success. This applies to all areas of life that involve human interactions. Since responsibility is part of the professional behavior of physicians and other health care professionals, it does not seem wrong to deal with it early on in training. At a training ward for nursing students, for example, it was found that they learn to take responsibility for patients and at the same time for their own learning by actively performing clinical activities [3]. In the medical profession, responsibility has been identified as one of three aspects of humanistic behavior, along with humility and the desire to achieve a high standard of professional behavior [4]. To achieve this attitude, self-reflection was identified as an essential factor in this study. Addressing the learning of self-reflection early in medical education and integrating it into medical curricula could be an important step towards familiarizing students with and practicing the need to take responsibility – for their own learning and for their subsequent medical practice. Indeed, it has been shown that the ability to self-reflect as an essential basis for taking responsibility does not develop automatically in medical school [5].

Thus, if undergraduate medical curricula and also postgraduate medical education are or should become more strongly aligned with the concept of entrustable professional activities (EPAs) [6], [7], [8], [9], methods for learning how to take responsibility must be considered at the same time, since reliable taking of responsibility forms the basis of entrustment. It is also important that EPAs should not be confused with competencies or skills [10]. That students are familiarized early on with reflecting on themselves and taking responsibility for their thoughts and actions is not only relevant for curricular design. It also plays a role in learning how to use naturopathy, complementary and integrative medicine, which Homberg et al. report on in a position paper in this issue [11] and especially for one's own actions in the context of a medical-experimental dissertation, for which a course was established by Griegel et al. and is presented in this issue [12]. In another position paper in this issue, Kaap-Fröhlich et al. present the current status and future perspectives of interprofessional education in the health professions [13]. In this form of learning, individual taking of responsibility and interprofessional exchange about it are of particular importance, as well as in communication courses, which had to be redesigned after 10 years of successful implementation due to the pandemic, which Schwär et al. report in this issue [14].

In these and other manuscripts in this issue of the GMS Journal for Medical Education, it can be found how essential learning to take responsibility is for all those working in the health professions. This central aspect of medical education and training in the health professions should receive even greater attention in the future. Since responsible action does not begin with training or medical work, the important aspect of taking responsibility could perhaps also come into focus in the future for admission procedures to enter undergraduate medical training.

## Competing interests

The author declares that she has no competing interests.
